# Virulence Plasmid (pYV)-Associated Expression of Phenotypic Virulent Determinants in Pathogenic *Yersinia* Species: A Convenient Method for Monitoring the Presence of pYV under Culture Conditions and Its Application for Isolation/Detection of *Yersinia pestis* in Food

**DOI:** 10.4061/2011/727313

**Published:** 2011-09-14

**Authors:** Saumya Bhaduri, James L. Smith

**Affiliations:** Molecular Characterization of Foodborne Pathogens Research Unit, Eastern Regional Research Center, Agricultural Research Service, U.S. Department of Agriculture, 600 East Mermaid Lane, Wyndmoor, PA 19038, USA

## Abstract

In *Yersinia pestis*, *Y. pseudotuberculosis*, and *Y. enterocolitica*, phenotypic expression of virulence plasmid (pYV: 70-kb)-associated genetic determinants may include low-calcium response (Lcr, pinpoint colony, size = 0.36 mm), colony morphology (size = 1.13 mm), crystal violet (CV) binding (dark-violet colony), Congo Red (CR) uptake (red pinpoint colony, size = 0.36 mm), autoagglutination (AA = cells agglutinate), and hydrophobicity (HP = clumping of cells). *Y. pseudotuberculosis* is chromosomally closely related to *Y. pestis;* whereas, *Y. enterocolitica* is chromosomally more distantly related to *Y. pestis* and *Y. pseudotuberculosis*. All three species demonstrate Lcr, CV binding, and CR uptake. The colony morphology/size, AA, and HP characteristics are expressed in both *Y. pseudotuberculosis* and *Y. enterocolitica* but not in *Y. pestis*. Congo red uptake in *Y. pestis* was demonstrated only on calcium-deficient CR magnesium oxalate tryptic soy agar (CR-MOX), whereas this phenotype was expressed on both CR-MOX and low-calcium agarose media in *Y. pseudotuberculosis* and *Y. enterocolitica*. These phenotypes were detectable at 37°C within 24 h in *Y. enterocolitica* and *Y. pseudotuberculosis* but did not appear until 48 h in *Y. pestis* due to its slower growth rate at 37°C. The pYV is unstable (i.e., easily lost under a variety of culture conditions) in all three species but is more unstable in *Y. pestis*. The specific CR uptake by *Y. pestis* in CR-MOX and the delayed time interval to express Lcr and CR uptake provide a means to differentiate *Y. pestis* from *Y. enterocolitica* and *Y. pseudotuberculosis*. These differences in pYV expression in *Y. pestis* can be used for its isolation and detection in food.

## 1. Introduction

The genus* Yersinia *consists of 11 species, but only *Y. pestis*, *Y*. *enterocolitica*, and *Y*. *pseudotuberculosis* are pathogenic to humans. *Yersinia pestis* is considered to be ancestrally related to *Y*. *pseudotuberculosis*; however, *Y. pseudotuberculosis* behaves phenotypically and clinically like *Y. enterocolitica *[1]. The three species are quite diverse in the diseases they cause; *Y*. *enterocolitica* and *Y*. *pseudotuberculosis* induce gastroenteritis when consumed in contaminated food and have been isolated from patients with diarrhea. *Yersinia pestis* is the agent of bubonic plague and can cause oropharyngeal plague as a result of the consumption of inadequately cooked goat and camel meat or handling of meat from infected animals [2–5]. The risk, morbidity, and mortality of contracting plague through the consumption of food deliberately contaminated with *Y*. *pestis* are currently unknown but potentially real. Furthermore, the identification of multidrug-resistant strains [6] and the potential use of this pathogen for the deliberate contamination of food could cause plague in large populations.

Three plasmids are involved in the virulence of *Y. pestis*: (a) pYV (virulence plasmid, 70-kb, Yops, type III secretion system), (b) pFra/pMT1 (96.2-kb, murine toxin: phospholipase, F1 capsule-like antigen), and (c) pCP1/pPst/pPla (9.6-kb, plasminogen activator) [8, 9]. Among these plasmids, the pYV-encoded type III secretion system (Yops) promotes cytotoxicity and the common symptoms of plague [8]. The pYV of all three species are of the same size and genetically highly conserved [8, 10–12]. It encodes the ability to target lymph tissues during infection and has genetic determinants essential for infection and overcoming host defense mechanisms [8, 10–12]. In the three species, carriage of pYV is responsible for the calcium-dependent growth phenotype at 37°C. The cultivation of pYV-bearing cells in low-calcium/calcium-deficient media elicits a Mg^2+^-dependent low-calcium response (Lcr), which results in the production of pYV-encoded virulence-associated antigens (V and W), and a series of released proteins (Yops). The low-calcium response is expressed phenotypically on solid media by the formation of pinpoint colonies [8, 10–12]. Furthermore, pYV in *Y*. *enterocolitica* has been correlated with several other *in vitro* characteristics, which are phenotypically expressed at 37°C. The well-characterized pYV-associated virulence determinants include colony morphology/size, Lcr, crystal violet (CV) binding, Congo red (CR) uptake, autoagglutination (AA), hydrophobicity (HP), mannose-resistant haemagglutination, expression of surface fibrillae, and serum resistance [11–13]. However, the expression of these physiological traits at 37°C also fosters the loss of pYV and the concomitant disappearance of the associated phenotypes. Since *Y*. *pestis* and *Y*. *pseudotuberculosis* have nearly identical chromosomal DNA sequences and are distantly related to pathogenic *Y*. *enterocolitica* [1, 12, 14], the purpose of this paper is to review whether the phenotypic characteristics induced by pYV are expressed in *Y*.* pestis* and *Y*. *pseudotuberculosis* and to determine the growth conditions required for the expression of these phenotypic characteristics. In addition, the detection and isolation of *Y. pestis *by monitoring the presence of pYV-encoded Lcr and CR-uptake virulence phenotypes are discussed.

## 2. Expression of pYV-Associated Phenotypic Virulence Determinants

**Figure 1:**
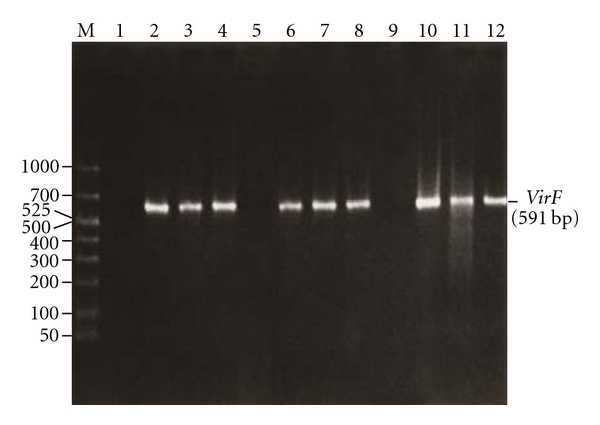
Confirmation of the presence of pYV in the original strains, cells in red pinpoint colonies, and cells in the white border around a red pinpoint colony from CR-MOX by PCR assay targeting a key regulatory gene *virF*, which encodes a transcriptional activator for the expression of pYV-encoded outer membrane protein Yop51. The primer pairs (5′-TCATGGCAGAACAGCAGTCAG-3′ and 5′-ACTCATCTTACCATTAAGAAG-3′) for detection of the *virF *gene (430- to 1020-nucleotide region) amplified a 591 base pair (bp) product from the virulence plasmid. Lane M, 50–1,000 bp ladder marker; lanes, 1, 5, and 9 showing the absence of 591-bp product in cells of the white borders of *Y*. *enterocolitica*, *Y*. *pseudotuberculosis*, and *Y*. *pestis,* respectively; lanes 2, 6, and 10 showing the presence of 591-bp product in the original strains of *Y*. *enterocolitica*, *Y*. *pseudotuberculosis*, and *Y*. *pestis* respectively before phenotypic evaluation; lanes 3, 7, and 11 showing the presence of 591-bp product in cells of the red pinpoint colonies of *Y*. *enterocolitica*, *Y*. *pseudotuberculosis*, and *Y*. *pestis,* respectively, and lanes 4, 8, and 12 showing the presence of 591-bp product within cells of red pinpoint colonies surrounded by white border of *Y*. *enterocolitica*, *Y*. *pseudotuberculosis*, and *Y*. *pestis* respectively [7].

In *Y. pestis, Y. pseudotuberculosis,* and *Y. enterocolitica,* the expression of phenotypic virulence characteristics is encoded by pYV [7]. A derivative of a clinical *Y. pestis* (KIM5: Kurdistan Iran man) strain lacking the chromosomal 102-kb Pgm locus (pigmentation), but harboring all three virulence plasmids (pYV, pFra/pMT1, and F1) [7, 9], was used for our study. The Pgm locus is only present in *Y*. *pestis*. This strain is conditionally virulent (a conditional mutant is only infectious if inoculated intravenously) and can be used in a BL2 laboratory facility [7, 9]. This strain shows CR-uptake in *Y*. *pestis* due to the presence of pYV; whereas, another derivative of a clinical strain of *Y. pestis*, the Kuma strain, contains the chromosomally encoded determinant, Pgm^+^ for CR-uptake but lacks pYV [7]. Clinical isolates of *Y*. *enterocolitica* (serotype O:3; strain GER) and *Y*. *pseudotuberculosis* (serotype O:1b; strain PB1/+) were also used in our study [7, 13]. The presence of pYV in *Y*. *pestis*, *Y*. *enterocolitica*, and* Y*. *pseudotuberculosis* was confirmed by a PCR assay targeting a key regulatory gene, *virF*, present on pYV (Figure 1, lanes 2, 6, and 10) [15]. The primers (5′-TCATGGCAGAACAGCAGTCAG-3′ and 5′-ACTCATCTTACCATTAAGAAG-3′) for the detection of the* virF* gene (430- to 1020-nucleotide region) amplified a 591-base pair (bp) sequence from the virulence plasmid [15]. *Yersinia pestis* Kuma strain did not show the presence of pYV by the PCR assay.

**Figure 2:**
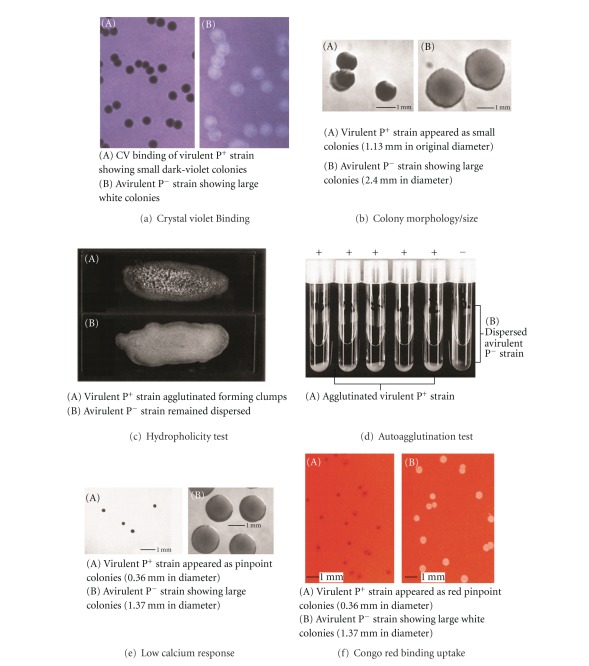
Evaluation of pYV-associated virulent phenotypes of pathogenic *Yersinia* species [7].

In our study, the pYV-negative derivatives (P^−^) of *Y*. *pestis *KIM5, *Y*. *pseudotuberculosis*, and *Y*. *enterocolitica* were obtained from large flat colonies, which emerged spontaneously from pYV-positive (P^+^) cultures growing at 37°C on brain heart infusion agarose with 238 **μ**M Ca^2+^ (BHO) [16] and were used as negative controls. The expression of pYV-encoded genetic determinants in *Y*. *pestis*, *Y*. *pseudotuberculosis,* and *Y*. *enterocolitica* was evaluated [7]. When P^+^ and P^−^ strains were cultivated at 37°C for 24–48 h on a low-calcium brain heart infusion agarose with 238 **μ**M Ca^2+^ (BHO), low-calcium tryptic soy broth agarose with 311 **μ**M Ca^2+^ (TSO), and calcium-deficient magnesium oxalate agar with tryptic soy agar (TSA) with 20% D-galactose, 0.25 M sodium oxalate, and 0.25 M magnesium chloride (MOX), the P^+^ cells of* Y*. *enterocolitica* and *Y*. *pseudotuberculosis* produced pinpoint colonies (0.36 mm in diameter; Figure 2(e)(A) at 24 h, whereas *Y*. *pestis* P^+^ formed pinpoint colonies at 48 h. The P^−^ cells from each representative strain formed much larger colonies (1.37 mm in diameter; Figure 2(e)(B). The size and colony morphology of each P^+^ strain when grown on 75 **μ**g/mL Congo red (CR) containing BHO (CR-BHO), TSO (CR-TSO), and 1% CR containing (CR-MOX) showed identical expression of Lcr as well as CR-uptake (0.36 mm diameter; Figure 2(f)(A) under all these conditions (Table 1). CR-uptake was demonstrated as bright red pinpoint colonies in *Y*. *enterocolitica* and *Y*. *pseudotuberculosis *on all three media (Table 2). However, CR-uptake of *Y*. *pestis* gave a less intense red color as compared to that of *Y*. *enterocolitica* and *Y*. *pseudotuberculosis* on CR-BHO and CR-TSO (Table 2). An increase of CR concentration in BHO and TSO to 100 **μ**g/mL, 150 **μ**g/mL, and 200 **μ**g/mL did not increase the color intensity of *Y*. *pestis* P^+^ colonies as compared to colonies of *Y*. *enterocolitica* and *Y*. *pseudotuberculosis*. On the basis of color contrast between the bacterial colony and the medium, CR-MOX was more suitable to show CR-uptake in *Y*. *pestis* as compared to CR-BHO (Table 2). The P^−^ cells from each representative strain failed to bind CR and formed much larger colonies (1.37 mm in diameter; Figure 2(f)(B). The difference of CR-uptake and the difference in timing of the expression of Lcr and CR-uptake in *Y*. *pestis* facilitate differentiating this species from *Y*. *pseudotuberculosis* and *Y*. *enterocolitica*. On calcium-adequate (1500 **μ**M) CR-BHA (brain heart infusion agar) and CR-TSA (tryptic soy agar), colonies of both P^+^ and P^−^ strains of *Y*. *pseudotuberculosis* and *Y*. *pestis* remained white or light orange similar to that reported for *Y*. *enterocolitica* (14, 17). The calcium concentration in CR-BHO (238 **μ**M Ca^2+^) and CR-TSO (311 **μ**M Ca^2+^) is relatively low, whereas, in CR-MOX, sodium oxalate is used to sequester calcium leading to a calcium-deficient medium. Thus, the CR-uptake in *Y*. *pestis* is more dependent on calcium depletion than that of *Y*. *enterocolitica* and *Y*. *pseudotuberculosis*. Moreover, *Y*. *pestis* Kuma, (Pgm^+^, pYV^−^) failed to bind CR on CR-MOX and formed large white or light orange colonies (1.37 mm in diameter) [7].

That the expression of CR-uptake on CR-MOX is specifically encoded by pYV was further confirmed using a number of derivatives of clinical strains of *Y. pestis* (CDC A1122, CO99.3015, Yokohama, P12, D1, D3, D5, D7, D9, D13, D17) containing Pgm but lacking the pYV [7, 10]. These Pgm^+^/pYV^−^ strains did not bind CR on CR-MOX. These observations indicate that the CR-uptake in *Y*. *pestis* grown on CR-MOX is associated with pYV. Thus, pYV-encoded CR-uptake is independent of Pgm^+^ and that the Pgm locus is not expressed on CR-MOX at 37°C. The CR phenotype is encoded by pYV only on calcium-depleted medium. Thus, CR-uptake in *Y*. *pestis* grown on CR-MOX is independent of chromosomally encoded CR binding virulence determinants (Pgm^+^) and is associated with the presence of pYV.

**Figure 3:**
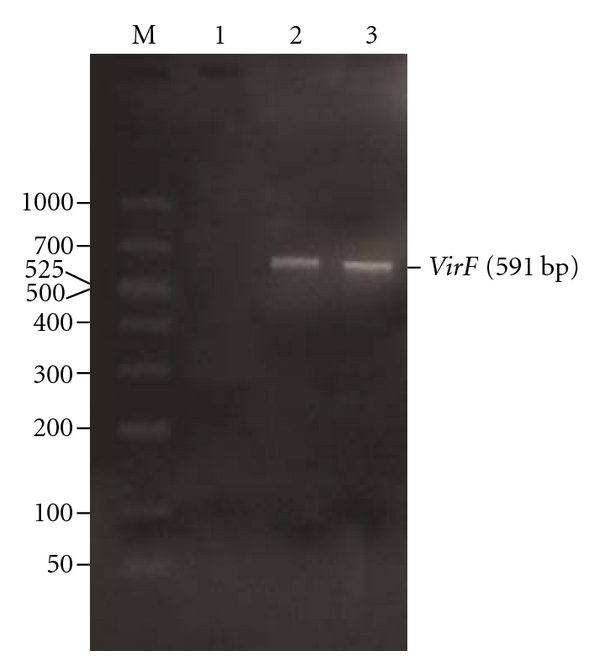
Detection of pYV in cells recovered from red pinpoint colony and subcultured in brain heart infusion broth at 28°C by PCR assay targeting *virF* gene of pYV. The primer pairs (5′-TCATGGCAGAACAGCAGTCAG-3′ and 5′-ACTCATCTTACCATTAAGAAG-3′) for detection of the *virF *gene (430- to 1020-nucleotide region) amplified a 591 base-pair (bp) product from the virulence plasmid. Lane M, 50–1,000 bp ladder marker; lane 1 showing the absence of 591-bp product in *Y*. *pestis*; lanes 2 and 3 showing the presence of 591-bp product in *Y*. *enterocolitica* and *Y*. *pseudotuberculosis,* respectively.

Another characteristic feature of the CR-uptake in P^+^ strains of *Y*. *enterocolitica* is the appearance of a white opaque circumference around the red center after 48 h of incubation at 37°C [17]. This characteristic colony type was also observed in *Y*. *pseudotuberculosis* after 48 h of incubation and in *Y*. *pestis* after 72 h of incubation. The timing of this colonial characteristic is another parameter that can be used for the identification of P^+^ strains of *Y*. *pseudotuberculosis* and *Y*. *pestis* [7, 17]. The cells in red pinpoint colonies (Figure 1, lanes 3, 7, and 11) and red centered colonies surrounded by a white border (Figure 1, lanes 4, 8, and 12) contained pYV in *Y*. *pseudotuberculosis* and *Y*. *pestis* similar to the cells reported in *Y*. *enterocolitica* [7, 17]. Cells in the surrounding white border (Figure 1, lanes 1, 5, and 9) do not contain pYV as demonstrated by PCR. When the pYV-bearing cells recovered from red pinpoint colonies were subcultured in BHI broth (brain infusion broth) at 28°C for 18 h, *Y*. *enterocolitica* and *Y*. *pseudotuberculosis *showed the presence of pYV by PCR (Figure 3, lanes 2 and 3) and pYV-associated phenotypic characteristics, while *Y*. *pestis* did not harbor pYV (Figure 3, lane 1) under the same conditions (Table 1). This showed that pYV is more stable in *Y*. *enterocolitica* and *Y*. *pseudotuberculosis* than in *Y*. *pestis*. Thus, CR uptake can also be used to isolate viable P^+^ cells in *Y*. *pseudotuberculosis* and *Y*. *enterocolitica* [7, 17–19]. 

The flooding of colonies of P^+^ strains on BHA, TSA, CR-BHO, CR-TSO, and CR-MOX grown at 37°C with CV solution at a concentration of 100  **μ**g/mL showed that P^+^ cells from all three *Yersinia* species bound CV and produced dark-violet colonies (Table 1; Figure 2(a)(A)). The P^−^ colonies did not bind CV and remained white (Figure 2(a)(B)). The CV- and CR-binding assays can effectively identify individual pYV-bearing colonies from a mixed culture of P^+^ and P^−^ strains [7, 17, 20]. The CR uptake is unrelated to CV binding; these two phenomena are independent since CV uptake is not related to Lcr.

The colony size of P^+^ cells in *Y*. *enterocolitica* and *Y*. *pseudotuberculosis* was smaller (1.13 mm in diameter; Figure 2(b)(A) than corresponding P^−^ cells when grown on BHA and TSA at 37°C (2.4 mm in diameter: Figure 2(a)(B) [16], whereas, P^+^ and P^−^ cells of *Y*. *pestis* were approximately the same size (1.3–1.4 mm SD ± 0.11 in diameter) at 37°C. This may be due to the fact that the optimum growth temperature of *Y*. *pestis* is 28°C (7, 9, 11, 14). Hydrophobicity by latex particle agglutination was positive (Figure 2(c)(A)) for pYV-bearing *Y*. *enterocolitica* and *Y*. *pseudotuberculosis* but negative for P^−^ cells (Figure 2(c)(B)). *Y*. *pestis* showed no HP when pregrown cells were tested from CR-BHO, CR-TSO, CR-MOX, BHA, and TSA (Table 1). Thus, HP of *Y*. *enterocolitica* and *Y*. *pseudotuberculosis* was expressed in low calcium, calcium-deficient, and calcium-adequate media, indicating that HP is also a non-Lcr property. 

The autoagglutination test in Eagle minimal medium supplemented with 10% fetal bovine serum was positive (Figure 2(d)(A)) for pYV-bearing *Y*. *enterocolitica* and *Y*. *pseudotuberculosis* but not for P^−^ cells (Figure 2(d)(B)). *Yersinia pestis* cultures failed to autoagglutinate (Table 1). In both the HP and AA tests, P^−^ strains were negative for the three species. The explanation for the absence of expression of the HP and AA phenotypic characteristics under the conditions described above in *Y*. *pestis* may be due to the lack of synthesis of pYV-associated surface factors essential for HP and AA or due to a structural/regulatory variability of pYV [21].

In conclusion, of the six pYV-associated phenotypes evaluated, only three phenotypes (Lcr, CR-uptake, and CV binding) were expressed in *Y*. *pestis*, while all six properties were expressed in *Y*. *enterocolitica* and *Y*. *pseudotuberculosis*. This differential expression of pYV-encoded phenotypes may be attributed to *in vitro* assay conditions although pYV is genetically highly conserved in all these species [6, 12, 14, 21]. Thus, the pYV-encoded phenotypes can be used as virulence markers for these pathogens [7, 10, 11, 13]. Although the chromosomal DNA sequence showed that *Y*. *pestis* and *Y*. *pseudotuberculosis* are nearly identical and closely related [1, 14], the latter exhibits the same expression of pYV-associated phenotypes as the more distantly related *Y*. *enterocolitica* and shows similar characteristics and clinical symptoms [1].

## 3. Procedure to Monitor the Presence of pYV in *Y. pestis* Cells during Storage and Culturing by Using the Lcr-CR-Uptake Techniques

The well-characterized pYV-associated virulence determinants can be used to determine plasmid maintenance, for isolation/detection, and as an indication of virulence for various serotypes of pYV-bearing *Y*. *enterocolitica* in food [11, 17–20, 22–25], as well as to determine the presence of pYV in *Y*. *pestis *and * Y*. *pseudotuberculosis *[7]. The pYV is unstable in all three pathogens, and the loss of pYV after cultivation or during food processing results in avirulent clones (not lethal to mice; do not cause plague) [7, 11, 13, 26–30]. Repeated transfer of cultures, extended storage at 4°C or −20°C, and laboratory manipulation, as well as subculturing of *Y. pestis* at temperatures >30°C leads to the loss of pYV [7, 29, 30]. Moreover, pYV is more unstable in *Y*. *pestis* (Figure 3, lane 1) than in of *Y*. *enterocolitica* and *Y*. *pseudotuberculosis* (Figure 3, lanes 2 and 3) [7, 29, 30]. The loss of pYV leads to the eventual overgrowth by cells lacking pYV and results in the loss of virulence and the concomitant disappearance of the pYV-associated virulence characteristics [7, 13, 25, 27, 28]. 

In a study on the growth of *Y*. *pestis* in ground beef, it was found that the cultures lost pYV during preparation of the inoculum [29]. It was not possible to maintain pYV in cells from the stock cultures using the standard procedures developed previously [13]. Thus, it was difficult to perform a study with *Y*. *pestis,* which reflected the actual behavior of pYV-bearing *Y*. *pestis*. In ground beef, the growth rates of pYV less cells were 0.096 and 0.287 CFU/h at 10 and 25°C, respectively; [30] whereas, for pYV-bearing cells, the growth rates were 0.057 CFU/h and 0.233 CFU/h at 10 and 25°C, respectively [29, 30]. The difference in growth rate between pYV-positive and pYV-negative strains of *Y*. *pestis* was more pronounced at lower temperatures. There was no growth of the pYV-bearing strain at 0 and 4°C as compared to the growth rates of pYV-negative strains of 0.003 and 0.016 CFU/h at 0 and 4°C, respectively, in ground beef [29]. Therefore, the lack of pYV leads to a faster growth rate and does not represent the true growth rate of the pYV-bearing strain. Hence, it is very important to maintain pYV in *Y*. *pestis* to properly study the growth behavior of a pYV-bearing strain in order to develop a growth model for this pathogen in food. The unstable nature of pYV in *Y*. *pestis* necessitates an examination for the presence of pYV and its virulence characteristics throughout laboratory manipulation and investigations. 

**Figure 4:**
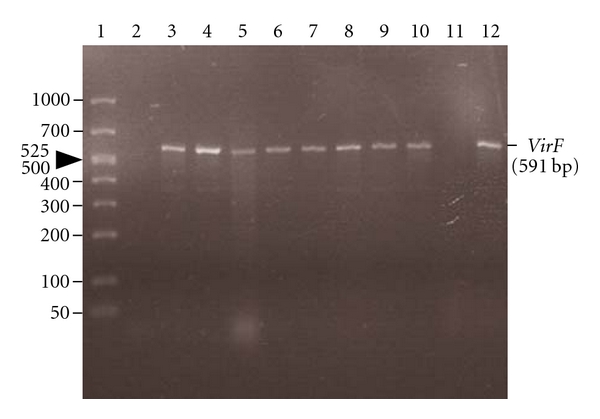
Confirmation of presence of pYV of *Y*. *pestis* in the original strain before subculturing, and CR-positive clones from CR-MOX, CR-BHO, and BHI broth using PCR assay targeting a key regulatory gene *virF* from pYV. The primer pairs (5′-TCATGGCAGAACAGCAGTCAG-3′ and 5′- ACTCATCTTACCATTAAGAAG-3′) for detection of the *virF *gene (430- to 1020-nucleotide region) amplified a 591-bp-product from the virulence plasmid. The Lcr-CR^+^ clones showed the presence of 591 bp products from pYV (lanes 2–10 and 12). Lane 1, 50–1,000 bp ladder marker; lane 2, negative control with no template; lane 3, original KIM5 strain as positive control; lanes 4, 5, Lcr-CR^+^ colonies from the CR-MOX and CR-BHO respectively (Figure 5; no. 1); lane 6 BHI broth (Figure 5; 1st passage; no. 2); lane 7** s**tock culture on CR-MOX (no. 3; 1st passage); lane 8 stock culture on CR-BHO (no. 3; 1st passage); lane 9 BHI broth (no. 4, 2nd passage from CR-MOX); lane BHI broth 10 (no. 4, 2nd passage from CR-BHO); lane 11 (no. 5, 2nd passage on CR-MOX) showing the absence of 591-bp product, and lane 12 (no. 5, 2nd passage on CR-BHO) [30].

**Figure 5:**
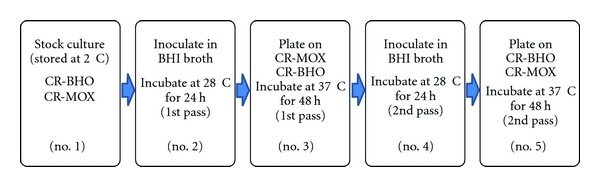
Confirmation of pYV in *Y. pestis*, [30].

Bhaduri et al. [30] developed a procedure to monitor the presence of pYV in *Y*. *pestis* cells during storage and culturing by using the Lcr-CR-binding techniques [7, 30], PCR assays, and the expression of pYV-associated virulence characteristics. It is essential to confirm the presence of pYV in the experimental culture by demonstrating that virulence-associated phenotypes were present and to confirm the presence of the pYV-encoded *virF *gene by a PCR assay (Figure 4, lane 3) [7, 15, 30]. The procedures for monitoring the presence of pYV and differentiating pYV positive clones from pYV-negative colonies during laboratory investigations are outlined in Table 3 [30]. As described in Table 3, the first step is to culture *Y. pestis* on CR-MOX and CR-BHO to isolate pYV-bearing clones from the frozen stock culture. The pYV-positive colonies appeared as red pinpoint colonies (0.36 mm in diameter) showing both Lcr and CR-uptake whereas pYV-negative colonies appeared as much larger white or orange colonies (1.37 mm in diameter) [7, 17, 30]. Colony morphology and CR-uptake were used to differentiate between pYV-positive clones and pYV-negative colonies. The Lcr and CR positive clones were further confirmed as pYV positive by the PCR assay (Figure 4, lane 4: CR-MOX and lane 5: CR-BHO) and by pYV-associated Lcr, CR uptake, and CV-binding phenotypes (see Table 3). These pYV-bearing clones were inoculated into BHI broth for the preparation of frozen and working stock cultures as described in Table 3. Before frozen storage and preparation of working stock cultures, the culture prepared in BHI broth at 28°C was tested for the presence of pYV and its virulence-associated phenotypes (Figure 4, lane 6). The Lcr-CR-positive clones on CR-MOX were used as working stock cultures and could be used for 15 days for laboratory studies. After that period of storage, the red pinpoint colonies of *Y. pestis* lost pYV (Figure 4, lane 11). The CR-BHO medium was also successfully used to ensure the selection of pYV in *Y*. *pestis* although CR-uptake was not as intense as on CR-MOX. The Lcr-CR positive clones were used as working stock cultures from CR-BHO and could be stored for 30 days at 2°C. To ensure the validity of this procedure for selecting pYV in *Y*. *pestis* cells, we also examined and monitored pYV stability during the subculturing of pYV-bearing cells in BHI broth, CR-MOX, and CR-BHO. *Yersinia pestis* from stock cultures stored at 2°C on CR-MOX and CR-BHO were subcultured as explained in Figure 5 [30]. The presence of pYV in *Y*. *pestis* cells in each medium and after each passage was monitored and confirmed at every step of culture transfer (no. 2–5) by the PCR assay for pYV and by the expression of pYV-associated phenotypic virulence characteristics, including Lcr, CR uptake, and CV binding. The PCR data for the presence of pYV is shown in Figure 1 (lanes 7–10). PCR results confirm that primers amplified a 591-base pair (bp) product from pYV (*virF* gene) for each phase of the culture as described above, and all PCR-positive clones on CR-MOX and CR-BHO showed their virulent phenotypic characteristics including Lcr, CR-uptake, and CV binding. The presence of the *virF* gene demonstrates the presence of pYV, which confers pYV-associated phenotypes.

In conclusion, the described procedure provides a method to ensure the selection of pYV-bearing strains of *Y*. *pestis* and for studying pYV-bearing *Y*. *pestis* without losing pYV during experimental procedures [30]. Although CR-BHO is a better medium for subculturing pYV-bearing *Y*. *pestis*, the pYV-bearing red pinpoint colonies are more easily detectable on CR-MOX due to more intense absorption of CR in the cells [7]. Hence, the use of CR-MOX for the preparation of stock cultures and to monitor the selection of pYV is recommended for investigation on the growth of pYV-bearing *Y*. *pestis* in food. Thus, this procedure will allow only the Lcr-CR-positive pYV-bearing clones to be used to study growth behavior, growth models, and related studies in food.

## 4. Application of CR-MOX for Isolation/Detection of *Yersinia pestis* in Food

*Yersinia pestis* can cause oropharyngeal plague as a result of the consumption or handling of meat from infected animals [2–5]. Thus, food intentionally contaminated by *Y. pestis *could have a significant role in the dissemination of human plague. Existing microbiological media designed for the selective isolation/detection of *Y*. *pestis* in food based on phenotypic analysis were found to be unsatisfactory. The purpose of this section is to review the development of alternative methods for identification/isolation of pYV-bearing *Y*. *pestis* based on the ability of *Y*. *pestis* to bind CR on calcium-depleted CR-MOX under specific conditions.

At present, the World Health Organization (WHO) [31] recommends the use of brain heart infusion (BHA) sheep blood agar and MacConkey agar for the isolation of *Y*. *pestis*. These growth media are suitable for sterile food; however, the isolation of *Y*. *pestis* from nonsterile foods is complicated by the presence of background flora competing for nutrients in the medium. Thus, the numerous colonies grown on these nonselective media require additional testing for the identification of the pathogen. MacConkey agar possesses a certain degree of selectivity; but the presence of CV and bile salt restricts the growth of *Y*. *pestis *[32]. *Y*. *pestis* strains exhibit slow or no growth *in vitro* on both cefsulodin-irgasan-novobiocin (CIN) agar and irgasan-nystatin agar [32] selective media when tested in our laboratory [7]. This may be due to the levels of selective substances used in this media. The colonies formed on selective media require further tests to identify them as *Y*. *pestis*. These tests are time consuming, costly, and labor intensive since a large number of presumptive colonies must be screened.

The calcium concentration in CR-BHO (234 **μ**M Ca^2+^) is relatively low, whereas in CR-MOX, sodium oxalate is used to sequester the calcium, making the medium calcium deficient [7, 16]. The comparison of CR uptake on calcium deficient CR-BHO and calcium-depleted CR-MOX among *Y*. *pestis, Y*. *pseudotuberculosis,* and *Y*. *enterocolitica* showed that this virulent phenotype is seen in pYV-positive strains of *Y. pestis* only when plated on calcium-depleted CR-MOX [7]. Thus, the CR uptake in *Y*. *pestis* is more dependent on calcium depletion than that of *Y*. *enterocolitica* and *Y*. *pseudotuberculosis*. Therefore, specific CR uptake on CR-MOX by *Y*. *pestis* can be used to differentiate* Y. pestis* from *Y. enterocolitica* and* Y. pseudotuberculosis* [7]. This would provide diagnostic value as follows: the suspected food samples are plated on CR-BHO and CR-MOX. If the colonies show CR uptake only on CR-MOX at 37°C after 48 h of cultivation, then those CR^+^ colonies can be isolated and identified as *Y*. *pestis* strains [7]. This technique will enhance the isolation/detection of *Y*. *pestis* strains in the presence of competing microflora by the proper selection of media and incubation times. The CR^+^ *Y*. *pestis* clones can be further confirmed by PCR targeting the *Y*. *pestis* specific plasmid-encoded plasminogen activator gene [33]. To show the specificity of CR uptake by *Y*. *pestis* on CR-MOX, several species of bacteria including a number of foodborne pathogens were tested. These non-*Yersinia* species did not form red pinpoint colonies and did not form a white border around the red center of the colony on CR-MOX [7, 17]. Furthermore, this method of isolation/detection for *Y*. *pestis* in food was verified by recovering the organism from artificially contaminated sterilized ground beef [29]. Thus, CR uptake on CR-MOX by *Y*. *pestis* provides a microbiological method for the isolation/detection of this pathogen. In conclusion, the specific CR uptake of *Y*. *pestis* in a calcium-deficient medium provides a screening medium to isolate, detect, and differentiate this pathogen from *Y*. *enterocolitica* and *Y*. *pseudotuberculosis*, and this method is also applicable to food.

## Figures and Tables

**Table 1:** Comparison of selected phenotypic expression of pYV-bearing *Y*. *enterocolitica, Y*. *pseudotuberculosis,* and* Y*. *pestis *(adapted from [7]).

Organism^a^	Strain	CM^b^	CV binding^c^	Lcr^d^	CR-uptake^e^	AA^f^	HP^g^	Plasmid^h^
*Y*. *enterocolitica *	GER	+	+	+	+	+	+	+
*Y*. *enterocolitica*-RE	GER	+	+	+	+	+	+	+
*Y*. *enterocolitica*-C	GER	−	−	−	−	−	−	−
*Y*. *pseudotuberculosis *	PB1/+	+	+	+	+	+	+	+
*Y*. *pseudotuberculosis*-RE	PB1/+	+	+	+	+	+	+	+
*Y*. * pseudotuberculosis*-C	PB1/+	−	−	−	−	−	−	−
*Y*. *pestis *	KIM5	−	+	+	+	−	−	+
*Y. pestis-*RE	KIM5	−	−	−	−	−	−	−
*Y*. *pestis*-C	KIM5	−	−	−	−	−	−	−
pYV-less *Y. pestis *	Kuma	−	−	−	−	−	−	−

^a^Cells recovered from red pinpoint colonies and subcultured in BHI broth at 28°C are designated as RE. The pYV-negative strains of *Y*. *enterocolitica*, *Y*. *pseudotuberculosis*, and *Y*. *pestis* are designated as C (cured).

^b^CM: colony morphology. On calcium-adequate BHA (1500 **μ**M Ca^2+^), and TSA (1400 **μ**M Ca^2+^) the P^+^ cells appeared as small colonies (1.13 mm in diameter) as compared to larger P^−^ colonies (2.4 mm in diameter).

^c^CV binding: crystal violet binding. The P^+^ cells appeared as small dark-violet colonies, and the P^−^ cells showed large white colonies on calcium-adequate BHA (1500 **μ**M Ca^2+^) and TSA (1400 **μ**M Ca^2+^), low-calcium CR-BHO (238 **μ**M Ca^2+^), CR-TSO (311 **μ**M Ca^2+^), and calcium-deficient CR-MOX.

^d^Lcr: low calcium response/calcium-dependent growth. P^+^ cells appeared as pinpoint colonies (0.36 in diameter), and P^−^ cells appeared large colonies (1.37 in diameter) on low-calcium CR-BHO (238 **μ**M Ca^2+^), CR-TSO (311 **μ**M Ca^2+^), and calcium-deficient CR-MOX.

^e^CR-Uptake: Congo red-uptake. The P^+^ cells appeared as red pinpoint colonies (0.36 in diameter), and the P^−^ cells appeared large white or light orange colonies (1.13 mm in diameter) on calcium-deficient CR-MOX.

^f^AA: autoagglutination. The P^+^ cells agglutinated. The P^−^ cells remained dispersed.

^g^HP: hydrophobicity by latex particles. The P^+^ cells formed clumps showing hydrophobicity. The P^−^ cells remained dispersed.

^h^Plasmid: presence of 70-kb pYV by PCR assay.

**Table 2:** Effect of media on CR-uptake in pYV-bearing *Y*. *enterocolitica*, *Y*. *pseudotuberculosis*, and *Y*. *pestis* (adapted from [7]).

Organism^a^	Strain	CR-BHO	CR-TSO	CR-MOX
*Y. enterocolitica*	GER	+	+	+
*Y. enterocolitica-*RE	GER	+	+	+
*Y. enterocolitica-*C	GER	−	−	−
*Y. pseudotuberculosis*	PB1/+	+	+	+
*Y. pseudotuberculosis-*RE	PB1/+	+	+	+
*Y. pseudotuberculosis*-C	PB1/+	−	−	−
*Y. pestis*	KIM5	−	−	+
*Y. pestis-*RE	KIM5	−	−	−
*Y*. *pestis*-C	KIM5	−	−	−
pYV-less* Y. pestis *	Kuma	−	−	−

^a^Cells recovered from red pinpoint colonies and subcultured in BHI broth at 28°C are designated as RE. The pYV-negative strains of *Y*. *enterocolitica Y*. *pseudotuberculosis,* and *Y*. *pestis* are designated as C (cured).

Low-calcium: CR-BHO (238 **μ**M Ca^2+^) and CR-TSO (311 **μ**M Ca^2+^). CR-MOX (calcium deficient).

**Table 3:** Isolation and maintenance of pYV in *Y. pestis *[30].

Day 1	

(i) Frozen stock cultures were streaked onto CR-MOX and CR-BHO.	
(ii) Plates were incubated at 37°C for 48 h for differentiation and isolation of pYV-bearing cells from pYV-less cells.	

Day 3	

(i) Using a stereomicroscope, red pinpoint colonies were examined to ensure Lcr and CR uptake. Using a sterile loop, 2-3 red pinpoint colonies were then inoculated into sterile 10 mL of BHI broth.	
(ii) The broth was inoculated and incubated at 28°C for 18–24 h.	

Day 4	

(i) The overnight culture was divided into three portions: frozen stock cultures, working stock cultures, and cells used for PCR assay and for expression of pYV-encoded virulent phenotypic characteristics including Lcr, CR uptake, and CV binding.	
(ii) Frozen stock cultures: 5 mL of overnight culture was mixed with equal portions of BHI broth and 20% glycerol and dispensed into 500 **μ**L portions for storage at *−*80°C.	
(iii) Working stock cultures: using a 10 **μ**L loop, cells were streaked on CRMOX and CR-BHO. The plates were incubated for 48 h at 37°C. Plates were then stored at 2°C for future use. Plates can be stored for 15 days for CR-MOX and 30 days for CR-BHO.	
(iv) PCR assay: 1 mL portion of cells was centrifuged, and DNA was prepared for PCR assay. Presence of pYV was confirmed by PCR assay targeting the *virF *gene in pYV.	
(v) The presence of pYV was also confirmed by demonstrating expression of phenotypic virulence characteristics including colonial morphology, CV binding, Lcr, and CR binding.	
